# Multi-omics analysis reveals GABAergic dysfunction after traumatic brainstem injury in rats

**DOI:** 10.3389/fnins.2022.1003300

**Published:** 2022-11-23

**Authors:** Qin Su, Qianling Chen, Zhigang Li, Jian Zhao, Lingyue Li, Luyao Xu, Bin Yang, Chao Liu

**Affiliations:** ^1^Guangzhou Forensic Science Institute, Guangzhou, China; ^2^Faculty of Forensic Medicine, Zhongshan School of Medicine, Sun Yat-sen University, Guangzhou, China; ^3^School of Forensic Medicine, Southern Medical University, Guangzhou, China

**Keywords:** traumatic brainstem injury, diffuse axonal injury, GABA, transcriptome, proteome, metabolome

## Abstract

**Background:**

Traumatic brainstem injury (TBSI) is one of the forms of brain injury and has a very high mortality rate. Understanding the molecular mechanism of injury can provide additional information for clinical treatment.

**Materials and methods:**

In this study, we detected transcriptome, proteomics, and metabolome expression changes in the brainstem of TBSI rats, and comprehensively analyzed the underlying mechanisms of TBSI.

**Results:**

After TBSI, there was significant diffuse axonal injury (DAI) in the brainstem of rats. A total of 579 genes, 70 proteins, and 183 metabolites showed significant changes in brainstem tissue. Through molecular function and pathway analysis, the differentially expressed genes, proteins, and metabolites of TBSI were mainly attributed to neural signal regulation, inflammation, neuroprotection, and immune system. In addition, a comprehensive analysis of transcripts, proteins, and metabolites showed that the genes, proteins, and metabolic pathways regulated in the brainstem after TBSI were involved in neuroactive ligand-receptor interaction. A variety of GCPR-regulated pathways were affected, especially GAGA’s corresponding receptors GABA_A_, GABA_B_, GABA_C_, and transporter GAT that were inhibited to varying degrees.

**Conclusion:**

This study provides insights into the development of a rapid diagnostic kit and making treatment strategies for TBSI.

## Introduction

Diffuse axonal injury (DAI) is one of the most common complications of traumatic brain injury (TBI), with an incidence of approximately 40∼50%, and a disease with extremely high mortality (Smith et al., 2003; Thomas and Dufour, 2009). In the 1950s, Strich (1956) conducted a seminal histopathological study showing that patients with post-traumatic disturbance of consciousness experience axonal damage in multiple brain regions, including the brainstem in addition to the cerebral hemispheres and subcortical white matter. Diffuse axonal damage in the brainstem has been identified as an association with adverse neurological outcomes, including long-term disturbance of consciousness and death (Adams et al., 1977; Firsching et al., 2002). Traumatic brainstem injury (TBSI) is an important manifestation of DAI in the brainstem region. The level of consciousness of patients with TBSI is more severely impaired, and the mortality rate is higher (Gentry et al., 1988). However, the molecular biological mechanism of TBSI is still less studied, and more information needs to be supplemented to assist clinicians in diagnosis and treatment.

Currently, the researches on TBSI are still dominated by clinical papers, and there is a lack of means to deeply study the molecular mechanism of TBSI (Crompton, 1971; Jang et al., 2016; Yamanishi et al., 2019). Animal models that mimic clinical conditions are useful tools for understanding TBSI-related pathophysiological changes. They can be used not only to study the molecular mechanisms underlying TBSI development and recovery, but also to evaluate therapeutic interventions. The mechanical impacting method can make the external mechanical force act directly on the skull under the premise of ensuring the same blow strength and action time, and can accurately hit the occipital tuberosity of rats, causing damage to the brainstem (Mychasiuk et al., 2014; Wu et al., 2015). The mechanical impacting method to establish an animal model of brainstem injury showed that the expression of GFAP was enhanced within 3 days after TBSI, which can be used to evaluate the degree of lesions in brain tissue after brainstem injury (Wu et al., 2015). The establishment of an animal model of brainstem injury has laid a good foundation for researchers to deeply explore the molecular mechanism of TBSI.

In recent years, multi-omics technologies including genomics, transcriptomics, metabolomics, and proteomics have been widely used to explore the mechanisms of various diseases (Ohashi et al., 2015), including but not limited to Alzheimer’s disease, human brain arteriovenous malformation, and other brain diseases (Wang et al., 2021; Winkler et al., 2022). In a previous study, Zhong et al. (2016) used transcriptomic techniques to observe changes of mRNA and lncRNA expression in the cortex of TBI model mice. Proteomics and metabolomics have also been applied to TBI biomarker identification (Azar et al., 2017; Banoei et al., 2018). Therefore, a comprehensive analysis combining transcripts, proteomes, and metabolites is expected to improve the understanding of the entire biological mechanism of TBSI to help clinicians identify strategies for timely intervention to combat TBSI.

In this study, we aimed to explore the full mechanism of TBSI. Understanding the complete mechanism will help to develop new strategies for the diagnosis and treatment of TBSI, especially in the early stages. In the serious brain trauma model, the 3-day group showed more differentially expressed proteins and metabolites than the 1-day group (Song et al., 2019), so rats 3 days after TBSI were selected for this study. We integrated transcript, proteome, and metabolite technologies to provide insights into the responses to TBSI and show that the data generated can be used to formulate new hypotheses and select candidate molecules for further functional studies.

## Materials and methods

### Animals

Male Sprague-Dawley (SD) rats with body weight between 220 g and 250 g used in this research were purchased from Beijing Vital River Laboratory Animal Technology Co., Ltd. Rats were maintained at a constant temperature (22 ± 1°C) and humidity (45∼60%) under specific-pathogen-free (SPF) conditions in Southern Medical University. The animals were subjected to experiments after an acclimatization period of 1 week. All experiments were approved by the Animal Experiments Ethics Committee of Guangdong Zhiyuan Biomedical Technology Co., Ltd. and in accordance with the Guide for Care and Use of Laboratory Animals.

### Construction of experimental animal model

Traumatic brainstem injury was performed on SD rats using a modified free-fall strike model (Mychasiuk et al., 2014), in which a height of 1 m and a 250 g stainless steel cylinder was chosen as the strike in our injury model (Supplementary Figure 1). Specifically, rats in the TBSI group were placed in an anesthesia induction chamber to induce anesthesia with 3% isoflurane until the rat’s corneal reflex disappeared and anesthesia was maintained with 2% isoflurane. The skin on the top of rats’ head was prepared and disinfected with iodophor. The scalp was cut along the midline to expose the periosteum, and the stainless steel spacer (diameter 1 cm, thickness 2 mm) was fixed in the middle of the sagittal suture with tissue glue (Supplementary Figure 1). The anesthesia device was then evacuated, and the rat was prone to be fixed on the sponge pad after being slightly awake so that the strike fell freely and hit the steel pad on the top of the rat’s head to produce a rapid hyperflexion movement of the head, and the stress responses of animals were measured immediately (BioNomadix MP160, BIOPAC, California, CA, United States). In the CTRL group, only the scalp was cut after using the same anesthesia and disinfection methods as that in the TBSI group, and the euthanasia method was the same as that in the TBSI group. Fourteen surviving rats in each group were anesthetized with sodium pentobarbital (100 mg/kg) and killed by decapitation 3 days later. The brainstem tissue was taken out and cut in half along the median sagittal plane. The brainstem tissues used for transcriptomic and proteomic analysis were from the same six animals while those used for histopathological and metabolomics analysis were from the other eight animals. All brainstem tissues were quick-frozen with liquid nitrogen and transferred to the –80°C refrigerator for storage except those used for histopathological analysis, which were fixed in 4% paraformaldehyde.

### Histopathological assay

To verify the success of the TBSI animal model, histopathological analysis was performed on paraffin-embedded brain tissue (8 rats per group). Bain stem tissues were fixed in 4% paraformaldehyde for 24 h. The cut surface was treated with a gradient of alcohol, xylene and paraffin, and the wax blocks were sectioned serially (5 μm) for hematoxylin and eosin (H&E) staining, silver staining (G1052, Servicebio, Wuhan, China) and immunohistochemistry (IHC) for GFAP. IHC was performed according to the protocol recommended in the instructions. Sections were dewaxed with xylene and hydrated in a series of ethanol solutions, followed by high temperature antigen repair by microwave in 0.01 M sodium citrate antigen repair solution (pH 6.0, AR0024, BOSTER, Wuhan, China) for 20 min. Subsequent blocking was performed according to the SP Rabbit and Mouse HRP Kit (CW2069, CWBIO, Taizhou, China) and after washing, primary antibody [GFAP (GA5) Mouse, #3670, Cell Signaling Technology, Boston, MA, United States] was diluted 1:200 with Antibody Diluent (P0262, Beyotime, Shanghai, China) and incubated overnight at 4°C. DAB color development was performed according to the kit the next day and the nuclei were stained with hematoxylin followed by xylene transparency and finally sealed with neutral gum.

### Transcriptomics assay

The total RNA of brainstem tissue was extracted using the TRIzol (thermofisher, 15596018, Waltham, MA, United States). Poly-A RNA was purified from total RNA with Dynabeads Oligo (dT) (Thermo Fisher, Waltham, MA, United States). The captured mRNA was segmented at high temperature using the Magnesium ion Fragmentation kit (NEBNextR RNA Fragmentation Module, CAT.e6150s, New England Biolabs, Ipswich, MA, United States). Reverse transcription of mRNA fragments was performed using reverse transcriptase (Invitrogen SuperScript™ II Reverse Transcriptase, cat.1896649, CA, United States). Indexed sequencing libraries were prepared using a commercial kit (ThruPLEX-Plasma Seq, Takara, Beijing, China) and sequenced using the illumina Novaseq™ 6000 (LC Bio Technology CO., Ltd. Hangzhou, China). Six biological replicates per group were included in this experiment. Sequencing reads were trimmed by removal of the adaptor sequences using Cutadapt^1^ (version 1.9) and aligned to the rat reference genome^2^ with HISAT2 (version 2.2.1). Transcript-level expression was calculated and normalized using StringTie (version 2.1.6) and Ballgown^3^ with the default parameters. Differentially expressed genes (DEGs) between TBSI and CTRL groups were estimated using R package DESeq2 (version 1.24.0). DEG identification was performed at the threshold of a fold-change > 2 or <0.5 and an adjusted *P*-value < 0.05. Kyoto Encyclopedia of Genes and Genomes (KEGG) enrichment analysis was performed using a hypergeometric distribution test by the R package “stats.” Relevant RNA sequencing raw data have been deposited in Genbank repository under accession number PRJNA 891128.

### A tandem mass tag proteomics assay

To obtain more significant results, a total of 12 samples from the TBSI and CTRL groups were selected for proteomic analysis, each group including six independent biological replicates. An appropriate amount of tissue was ground in liquid nitrogen and lysis buffer (8M Urea, 1% protease inhibitor cocktail) was added. Then the samples were lysed by sonication on ice and left on ice for 30 min. Centrifuge at 20,000 *g* at 4°C for 10 min, and collect the supernatant. The protein concentration was detected using the BCA kit. 100 μg of protein from each sample was added DTT to reduce disulfide bonds, and maintained at 30°C for 60 min. Then IAM was added to block free sulfhydryl groups at room temperature for 45 min in the dark. The protein was precipitated by acetone. TEAB was added and the protein precipitate was dissolved by sonication on ice. Following this, trypsin was added to digest overnight at 37°C. The acidified peptides were desalted with C18 SPE column, and the eluted peptides were drained with a vacuum concentrator. 100 μg of peptides were taken out from each sample for labeling according to the instructions of the TMT reagent. The dried labeled peptides were redissolved in liquid A. 50 μg peptides from each sample were mixed and centrifuged at 20,000 *g* for 2 min. The supernatant was loaded into C18 column, and the reverse gradient separation was carried out by conventional high performance liquid chromatography under alkaline condition. According to the peak range displayed by the UV lamp, the elution peptide components were selected, and drained by vacuum concentrator. Each component was dissolved in liquid A, centrifuged at 20,000 *g* for 2 min, and the supernatant was transferred to the top sample bottle. Each component was analyzed by liquid mass spectrometry for 1 h with sample loading of about 2 μg. Raw files of MS were retrieved directly using Maxquant (Version 1.5.2.8).

### Untargeted metabolomics assay

One hundred twenty milligrams tissue of each sample was ground with liquid nitrogen, and 120 μL 50% methanol was added to mix thoroughly to extract the metabolites. The mixture was allowed to sit at room temperature for 10 min and transferred to −20°C overnight to precipitate the protein and then centrifuged at 4,000 *g* for 20 min. Metabolite extract of supernatant was taken to a 96-well plate for machine detection. 10 μL of diluent was equally taken from each sample and mixed into a quality control (QC) sample to test the stability of the method. Liquid chromatography mass spectrometry (LC-MS) was performed on a Thermo Scientific UltiMate 3000 HPLC. An ACQUITY UPLC BEH C18 column (2.1 × 100 mm, 1.8 μm, Waters, Wilmslow, United Kingdom) was used for the reversed phase separation and maintained at 35°C. The mobile phase was consisted of 0.1% formic acid (mobile phase A) in water and 0.1% formic acid in acetonitrile (mobile phase B) running at a flow rate of 0.4 ml/min. Gradient elution conditions were set as follows: 0∼0.5 min, 5% B; 0.5∼7 min, 5–100% B; 7∼8 min, 100% B; 8∼8.1 min, 100–5% B; 8.1∼10 min, 5% B. The injection volume for each sample was 4 μl. MS was performed on a Q-Exactive (Thermo Fisher Scientific), which was operated in both positive and negative ion modes. To evaluate the stability of the LC-MS during the whole acquisition, QC was acquired after every 10 samples. Metabolomic data were analyzed using the XCMS software package^4^. Each ion was identified by combining retention time (RT) and m/z data. Intensities of each peak were recorded and a 3D matrix containing arbitrarily assigned peak indices (retention time-m/z pairs), sample names (observations), and ion intensity information (variables) was generated. The KEGG database was used to annotate the metabolites by matching the exact molecular mass data (m/z) of samples with those from database. Besides, an in-house fragment spectrum library of metabolites was used to identify the metabolite. The intensity of peak data was further preprocessed by metaX. Those features that were detected in less than 50% of QC samples or 80% of biological samples were removed. The remaining peaks with missing values were imputed with the k-nearest neighbor algorithm to further improve the data quality. Principal-component analysis (PCA) was performed for outlier detection and batch effects evaluation with the preprocessed data set.

### Systems biology analysis

To analyze the correlation between the transcriptome and proteome, the relationship between DEGs and DEPs were exhibited by a quadrant map. For integration analysis including correlations between the transcriptome, proteome and metabolome, DEGs, DEPs, and differential metabolites, respectively, were mapped to the KEGG database. Metabolome, proteome, and transcriptome relationships were visualized using Cytoscape (version 3.4.0).

### Data analysis

Statistical analysis was performed using R language (version 4.1.2). All data were presented as the mean ± SEM and were analyzed by *T*-tests. A *P*-value < 0.05 was considered statistically significant, *P < 0.05, ^**^P < 0.01, ^****^P < 0.0001. Transcripts were considered to be differentially expressed at fold change > 2 or <0.5 and were statistically significant (*Q* value < 0.05). Proteins upregulated or downregulated (fold change ≥ 1.2 or ≤0.83, respectively, *p* < 0.05) were defined as differentially expressed proteins (DEPs). Metabolites were considered statistically significant if variable influence on projection (VIP) ≥ 1.2 and *P*-value < 0.05 in the PLS-DA model.

## Results

### Stress responses

**FIGURE 1:**
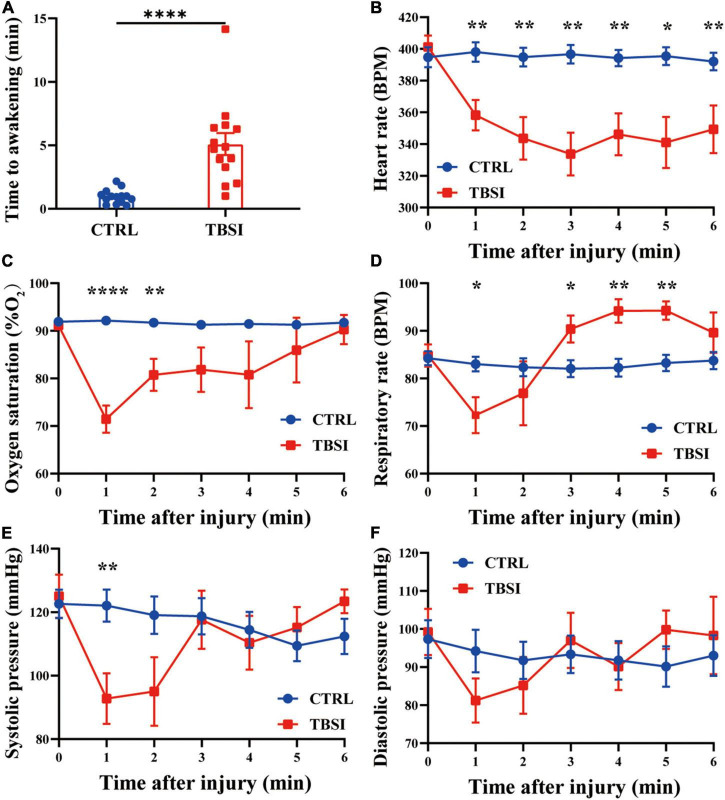
Stress response. **(A)** Time to awakening. **(B)** Heart rate. **(C)** Percutaneous arterial oxygen saturation. **(D)** Respiratory rate. **(E)** Systolic pressure. **(F)** Diastolic pressure. Results are mean ± SEM of 14 rats in each group. *T*-tests, *P < 0.05, **P < 0.01, ****P < 0.0001.

In this study, 3 animals died within 10 min after injury in the TBSI group, with a mortality rate of 17.65%, while the other rats immediately fell into unconsciousness (5.10 ± 3.23 min, Figure 1A). The time to awakening was significantly longer than the CTRL group. At the same time, other stress responses were recorded within 6 min after injury. It was found that heart rate and SpO2 (%) in the TBSI group decreased continuously after injury, and SpO2 (%) tended to normal at 6 min (Figures 1B,C). The respiratory rate decreased significantly 1 min after injury, then increased and leveled off (Figure 1D). Systolic blood pressure and diastolic blood pressure had similar changes, but the former decreased more significantly 1 min after injury and leveled off 3 min later (Figures 1E,F). These results suggest that TBSI rats experienced multiple stress responses, including varying degrees of disturbance of consciousness, transient circulatory, and respiratory dysfunction. In addition, compensatory recovery was achieved in a certain period of time, all of which are similar to TBSI in human body. Therefore, it is confirmed that TBSI is caused by this modeling method to a certain extent.

### Pathological analysis of traumatic brainstem injury model

**FIGURE 2:**
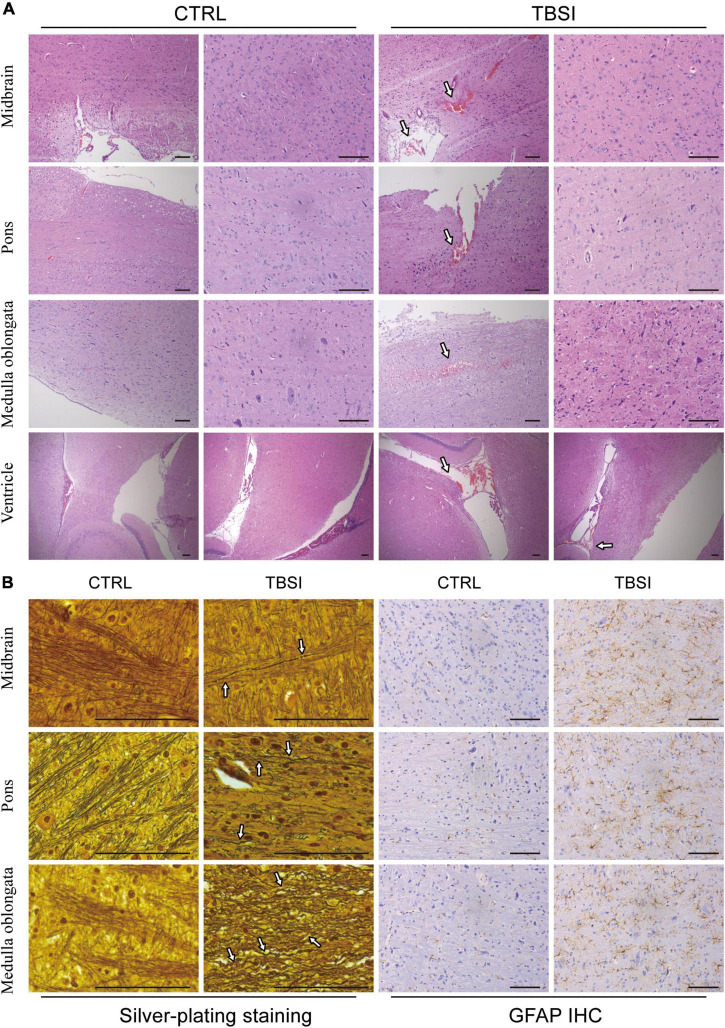
Brainstem pathological analysis. **(A)** Hematoxylin and eosin (H&E) staining. Arrows point to the areas of bleeding. **(B)** Silver staining (Left) and GFAP staining (Right). Arrows point to disordered axons. Scale bar = 200 μm.

To further evaluate the construction of our TBSI model, gross anatomy, H&E staining, silver staining, and GFAP IHC analysis were used to illustrate the results from different perspectives. The results of gross anatomy showed that subarachnoid hemorrhage of different degrees was found in the brainstem of TBSI group, while no obvious abnormality was found in the CTRL group (Supplementary Figure 2). By H&E staining, it was found that rats in the TBSI group not only had ventricular hemorrhage of varying degrees and multiple focal hemorrhages in the brainstem parenchyma, but also the peripheral gaps of nerve cells and blood vessels in the parenchyma were widened. In the CTRL group, the morphology and structure were normal and no obvious pathological changes were observed (Figure 2A). The result of silver staining showed that the brainstem axons of the CTRL group were arranged neatly and uniformly, while the axonal arrangement of the TBSI group was disordered, diffusely fractured, obviously swollen, twisted like an earthworm, and contracted into a spherical shape (Figure 2B). GFAP immunohistochemical staining was used to observe astrocyte responses after TBSI. The result showed that compared with the CTRL group, astrocytes in the TBSI group increased in number, volume, branching, and cytoplasmic staining (Figure 2B). These results confirm that the TBSI model of this study is successful and that DAI can be induced in the brainstem after TBSI.

### Effects of traumatic brainstem injury on the brainstem transcriptome

**FIGURE 3:**
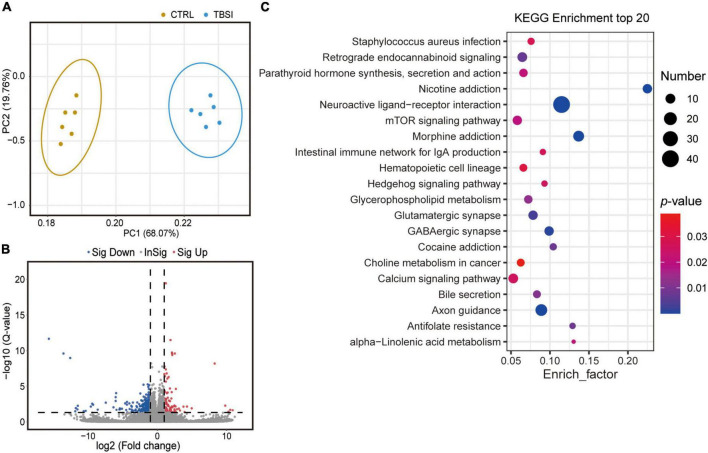
Transcriptomic data analysis of rat brainstem. **(A)** Principal component analysis (PCA) of RNA-seq data. The dots represent biological replicates. **(B)** Volcano plot showing relative transcript abundance. Gray dots indicate no statistically significant differences in gene expression. Red dots represent up-regulated genes and blue dots represent statistically significant down-regulated genes. **(C)** KEGG pathway analysis of the top 20 outcomes of DEGs identified between the TBSI and CTRL groups.

To determine gene expression changes, RNA-seq analysis was used to investigate differentially expressed genes (DEGs) between the TBSI and CTRL groups. PCA results showed that transcripts were significantly different (Figure 3A) and a total of 24,830 genes were detected. DEGs were displayed in the volcano plot, and there were large differences in differentially enriched genes between the TBSI and CTRL groups (Figure 3B). In total, 569 genes were found to be differentially expressed, of which 101 and 468 genes were up- and down-regulated, respectively. Next, we investigated the potential biological functions of DEGs. The top 20 KEGG pathway enrichment analysis results (Figure 3C; Supplementary Table 1) showed that the altered genes were mainly involved in neuroactive ligand-receptor interaction, nicotine addiction, morphine addiction, axon guidance, GABAergic synapse, and glutamatergic synapse. Some of these signaling pathways are known for their key roles in the regulation of neural signaling. These results suggest that distinct pathways are triggered after TBSI.

### Effect of traumatic brainstem injury on brainstem proteomic analysis

**FIGURE 4:**
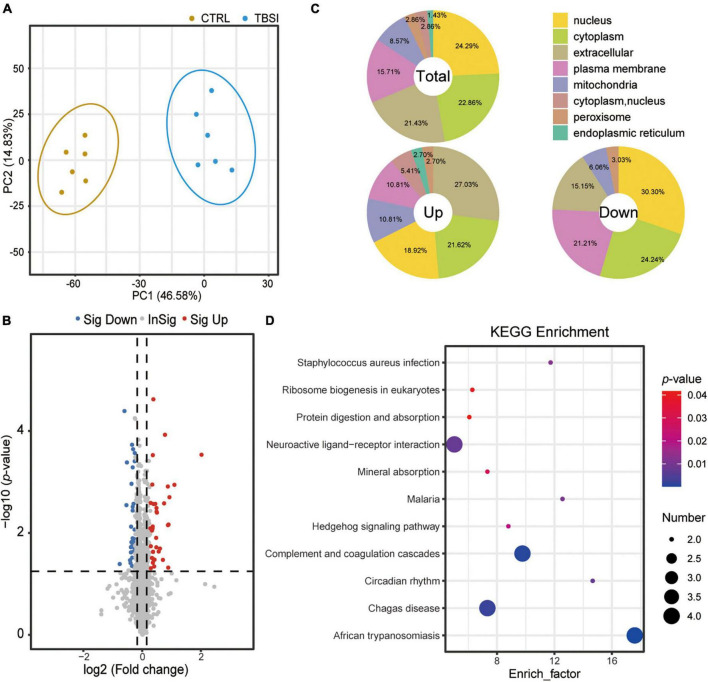
Analysis of proteomic data in rat brainstem. **(A)** PCA of proteomic data. The dots represent biological replicates. **(B)** Volcano plot showing relative protein abundance. Gray dots indicate no statistically significant differences in protein expression. Red dots represent up-regulated proteins and blue dots represent statistically significant down-regulated proteins. **(C)** Location of the total, up-, and down-regulated DEPs. Total: 70 quantifiable proteins. Up: 31 upregulated proteins. Down: 39 down-regulated proteins. **(D)** KEGG pathway analysis results of DEPs identified between the TBSI and CTRL groups.

A total of 67,184 peptides were detected by proteomic analysis, of which 61,126 unique peptides were derived from 6,773 proteins, and 6,274 proteins were quantitatively analyzed. PCA showed that the proteins differed significantly between the TBSI and CTRL groups (Figure 4A). Compared with the CTRL group, the TBSI group had 31 up- and 39 down-regulated proteins (Figure 4B). The results of subcellular structure annotation classification demonstrated that DEPs were mainly enriched in nucleus, cytoplasm, extracellular, and plasma membrane (Figure 4C). KEGG enrichment analysis was performed on DEPs (Figure 4D; Supplementary Table 2), and they were mainly involved in African trypanosomiasis, complement and coagulation cascades, Chagas disease, neuroactive ligand-receptor interaction, circadian rhythm, malaria, staphylococcus aureus infection, and Hedgehog signaling pathway. These results provide new insights for studying the further mechanism of TBSI in rats.

### Effect of traumatic brainstem injury on brainstem metabolomic analysis

**FIGURE 5:**
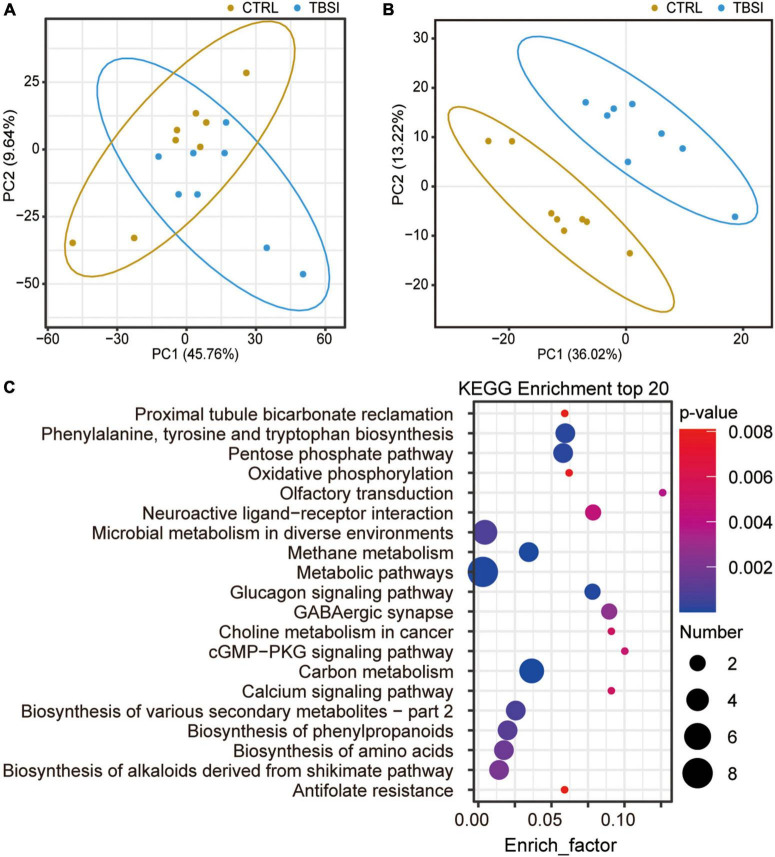
Characterization of brainstem metabolomics. **(A)** PCA analysis of metabolic profiling results. The dots represent biological replicates from each group. **(B)** Score plot of PLS-DA model for classification of TBSI and CTRL groups. **(C)** KEGG analysis of potentially perturbed metabolic pathways associated with potential biomarkers in the TBSI group.

A total of 2,950 metabolites were identified in this experiment (1,917 and 1,033 metabolites were identified in positive and negative ion mode, respectively): organic acids and derivatives (198, 37.08%), lipids and lipid-like molecules (109, 20.41%), organoheterocyclic compounds (77, 14.42%), organic oxygen compounds (38, 7.12%), benzenoids (33, 7.17%), nucleosides, nucleotides, and analogues (29, 5.43%), organic nitrogen compounds (22, 4.12%), phenylpropanoids and polyketides (16, 3.00%), others (12, 2.25%) (Supplementary Figure 3). PCA showed that the TBSI and CTRL groups could not be distinguished effectively after clustering (Figure 5A). To obtain a more specific statistical analysis, a PLS-DA model was employed. The intercept Q2 was −0.5451, indicating that there was no over-fitting of the model and the analysis of differential metabolites was accurate (Supplementary Figure 4). The PLS-DA score plot demonstrated a clear separation and a clustered pattern for metabolism among the two groups (Figure 5B). Through the calculation of VIP, differential metabolites (VIP ≥ 1.2, and *P*-value < 0.05) were screened. Compared with the CTRL group, the TBSI group had 183 differential metabolites in positive ion mode (48 up-regulated, 135 down-regulated), and 71 differential metabolites in negative ion mode (16 up-regulated, 55 down-regulated). The top 20 results of KEGG pathway enrichment analysis showed that the differential metabolites were mainly involved in carbon metabolism, methane metabolism, metabolic pathways, glucagon signaling pathway, phenylalanine, tyrosine and tryptophan biosynthesis, GABAergic synapse and neuroactive ligand-receptor interaction (Figure 5C; Supplementary Table 3). Most of the differential metabolites are enriched in metabolic pathways, which certified that the metabolic pathways are closely related with TBSI. Above all, these pathways are obviously affected by TBSI and could be considered as candidates for understanding the mechanism of TBSI.

### Correlative functional analysis of transcriptome, proteome, and metabolome datasets

**FIGURE 6:**
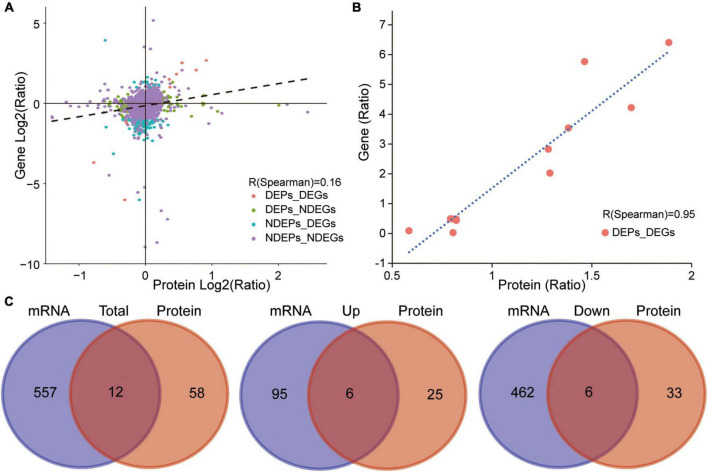
Correlation between transcriptome and proteome. **(A)** Correlation analysis of transcriptome and proteome expression (TBSI vs. CTRL). **(B)** Correlation analysis of DEGs and DEPs (TBSI vs. CTRL). **(C)** Summary of overlapping DEG and DEP numbers in TBSI and CTRL groups (Left). Up-regulated overlapping DEG and DEP numbers (Middle). Down-regulated overlapping DEG and DEP numbers (Right).

**FIGURE 7:**
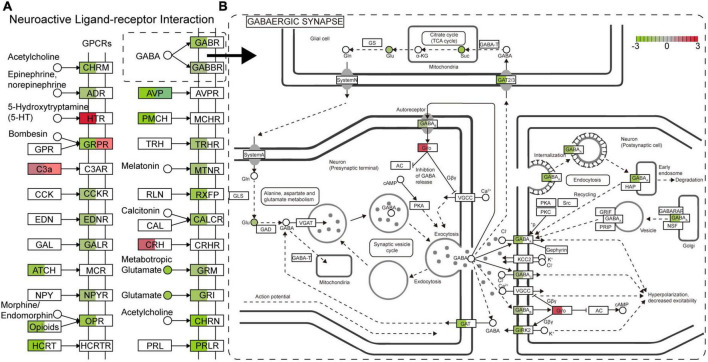
The main enrichment pathway neurologyactive ligand-receptor interaction **(A)** and GABAergic synapse (B) in the TBSI group. Red: up-regulated; green: down-regulated. □ is a gene or protein, the color shows the expression change of the gene (Left) or protein (Right). ° is a metabolite.

As described above, a number of genes, proteins, and metabolites were detected, which deserve further attention and in-depth functional study and validation as TBSI biomarkers. To narrow the possible candidate biomarkers, we further performed a multi-omic analysis. Spearman correlation analysis showed that the correlation coefficient between transcript and protein expression level was 0.16, while for DEGs and DEPs, the correlation coefficient was 0.95, and the number of genes that could be associated was 12 (Figures 6A,B). Among them, 6 genes and their corresponding proteins were up-regulated: MT1M, MT1, LGALS3, C1QB, HSPB1, GFAP, and the other 6 were down-regulated: SULT1D1, PCDH10, FAM189A1, AVP, TTR, PDLIM5 (Figure 6C). The results of KEGG pathway analysis showed that the neurologyactive ligand-receptor interaction pathway was enriched at the transcriptome, proteome and metabolome levels simultaneously. In this pathway, a variety of GPCRs or their ligands were affected, including CHRM, ADR, HTR, GRPR, C3AR, CCKR, EDNR, GALR, MCR, NPYR, OPR, HCRTR, GABR, GABBR, AVPR, MCHR, TRHR, MTNR, RXFP, CALCR, CRHR, GRM, GRIN, CHRN, and PRLR (Figure 7). Among them, multiple receptors of GABA were down-regulated. Analysis of the GABAergic synapse pathway (Figure 7) revealed that multiple GABA receptors and transporter were down-regulated, including GABA_A_, GABA_B_, GABAC, and GAT. These results suggest that GABAergic pathways in the brainstem are extensively inhibited after TBSI. Taken together, the integrated multiomics analysis identified multiple genes and pathways that are potentially linked to TBSI, all of which need to be further validated, either as single markers or as marker sets for further applications.

## Discussion

This study investigated the transcriptomic, proteomic, and metabolomic changes induced by brainstem DAI after TBSI. Brainstem DAI was induced in adult male SD rats by using a modified free-fall striking model. The axonal pathological changes that occurred were effectively demonstrated by H&E staining, silver staining and GFAP IHC staining 3 days after TBSI. Understanding the molecular mechanism changes in the brainstem after TBSI will help develop new strategies to diagnose and treat TBSI.

Currently, RNA-seq technology has been widely used for systematic exploration of transcriptome information with high throughput and large depth of coverage (Byrne et al., 2019). Given the exploration of TBSI-induced DAI-related gene expression changes, RNA-seq analysis is a particularly useful tool that can provide insights into DAI-related mechanisms. The results indicated that the DEGs could be attributed to multiple stress response pathways, including signaling, inflammation, neuroprotection, and the immune system, etc. Neuroactive ligand-receptor interaction has the strongest correlation with DAI induced by TBSI, and the regulatory functions of multiple GPCRs in the pathway are affected. Neuroactive ligand-receptor interactions are often associated with neurological diseases such as Parkinson’s disease, encephalopathy, and encephalitis (Lin et al., 2015; Kelly et al., 2020; Zhang et al., 2022). In traumatic brain injury (TBI), the pathway neuroactive ligand-receptor interaction is also affected (Huang et al., 2019; Zhou et al., 2019; Sachdev et al., 2022). In addition to being enriched in neuroactive ligand-receptor interaction, DEGs are also enriched in other neuronal signaling pathways, such as axon guidance, GABAergic synapse, glutamatergic synapse, retrograde endocannabinoid signaling, calcium signaling pathway, etc. The pathways enriched by DEGs are also related to addiction, such as nicotine addiction, morphine addiction, and cocaine addiction. DEGs related to these pathways were mostly low-expressed in the TBSI group. It has been reported that brain injury (e.g., stroke) leads to remission of addiction in patients (Abdolahi et al., 2015; Joutsa et al., 2022), which is consistent with our results. TBSI apparently causes pathological symptoms such as fractures and inflammation. Parathyroid hormone (PTH) is one of the three key hormones that regulate calcium and phosphate homeostasis, so enrichment of the pathway parathyroid hormone synthesis, secretion, and action can promote the recovery of TBSI-induced skull fractures (Bajwa et al., 2018). Hedgehog signaling pathway, mTOR signaling pathway, and hematopoietic cell lineage have been reported to protect neurons in disordered environments (Grochowski et al., 2018; Xu et al., 2018; Yin et al., 2020). HSPB1 showed high expression in the transcriptome and proteome when the TBSI group was compared with the CTRL group. The main function of HSPB1 is to provide thermotolerance *in vivo*, cytoprotection, and support of cell survival under stress conditions (Miller and Fort, 2018). Inflammatory factors activate microglia and astrocytes to generate an inflammatory immune response, leading to reorganization of neural circuits in the brainstem (Burda et al., 2016). We found that immune-related pathways and genes such as the C1/C3 complement system, MHC-II, FcγR, etc., in staphylococcus aureus infection and intestinal immune network for IgA production pathway are activated. We also observed that GFAP in the brainstem of the TBSI group was up-regulated in both transcriptome and proteome, which is consistent with the phenomenon of astrogliosis observed in pathological sections. GFAP is a marker of astrocyte activation (Jurga et al., 2021). Previous study has confirmed that the formation of a large number of astrocyte scars after traumatic brain injury is formed by new astrocytes rather than existing activated cells or migrating cells (Kernie et al., 2001). Glycerophospholipid metabolism was found to play a key role in the pathophysiological mechanism of axonal damage in brain DAI (Zhang et al., 2018) and it was also enriched in brainstem DAI. In conclusion, TBSI-induced DAI mainly causes inflammation and immune system damage as well as disturbance of neuronal signaling regulatory pathways, while multiple pathways generated stress responses to protect neurons and eliminate inflammation. Transcriptome data can serve as a starting point for further mechanistic analysis and may represent candidates for biomarker exploration.

The proteome in this study quantified more than 6,274 proteins in the brainstem of the TBSI group. The results showed that the correlation between the overall proteome and transcriptome was poor, with a correlation coefficient R of only 0.16. This may be due to the differential lifetimes of mRNAs compared with proteins, protein transport and posttranscriptional modification (Perl et al., 2017). However, the correlation between DEPs and DEGs is very high, and the correlation coefficient R is as high as 0.95, indicating that there was a positive correlation between DEGs and DEPs. The KEGG pathway enrichment analysis showed that only 11 pathways were enriched, which may be related to the low number of DEPs. Three of them are the same pathways that DEGs are enriched to, including neuroactive ligand-receptor interaction, staphylococcus aureus infection, and Hedgehog signaling pathway. In addition, the enriched complement and coagulation cascades pathway is also one of the important pathways of complement cascade activation in brain injury (Hammad et al., 2018). The results of proteomics further validated the analysis of transcriptomic data. We detected 2,950 metabolites in metabolomics, of which 254 metabolites were significantly changed in the brainstem of the TBSI group. Three pathways coexisted in these differential metabolites and DEGs, including GABAergic synapse, neuroactive ligand-receptor interaction and calcium signaling pathway, all of which were involved in the regulation of neural signaling. In addition, the glucagon signaling pathway is also enriched in the metabolome, and it has been reported that glucagon-like peptide-1 (GLP-1) can reduce neuroinflammation induced by brain injury and play a neuroprotective role (Huang et al., 2020; Yang et al., 2022). The differential metabolites identified in the TBSI group were also involved in phenylalanine, tyrosine and tryptophan biosynthesis. Tryptophan and phenylalanine can be components of protein synthesis and provide higher nutrient levels, and are also essential substrates for the synthesis of the neurotransmitters serotonin and dopamine, respectively (Fernstrom and Fernstrom, 2007; Durham et al., 2017).

Multi-omics techniques have emerged as powerful tools for molecular analysis and elucidation of complex systems biology (Nicora et al., 2020). Neuroactive ligand-receptor interaction pathway was enriched in transcriptome, proteome and metabolome. In the TBSI group, multiple GABA receptors GABA_A_, GABA_B_, and GABA_C_, as well as the transporter GAT were inhibited. GABA regulates excitatory pathways in the brain, and loss of GABA-producing cells disrupts the balance of excitation and inhibition, leading to further cellular damage and apoptosis (Guerriero et al., 2015). In the brainstem of the TBSI group, the activity of GABA was inhibited and the excitability of neural circuits could not be balanced, which is consistent with the findings in the acute phase of traumatic brain injury (McGuire et al., 2019).

Since the results of this study mainly come from rat experiments, and animal experimental studies are sometimes incomplete, it is not reliable to extrapolate the results obtained through animal models to animals of other ages or other species, and further studies are needed to confirm.

In conclusion, the current comprehensive application of transcriptome, proteome, and metabolome analysis has potential significance for understanding the molecular mechanism of TBSI-induced DAI, and provides new ideas for the diagnosis and treatment of TBSI.

## Data availability statement

The datasets presented in this study can be found in online repositories. The names of the repository/repositories and accession number(s) can be found in the article/Supplementary material.

## Ethics statement

This animal study was reviewed and approved by Animal Experiments Ethics Committee of Guangdong Zhiyuan Biomedical Technology Co., Ltd.

## Author contributions

QS, BY, and CL designed the study. QC, ZL, and LL collected the brainstem materials of rats. QS and LX analyzed the RNA-seq data. QS, JZ, and CL wrote the manuscript, with the help from BY. JZ checked the reproducibility of the results. All authors contributed to interpretation of the results, revised the manuscript, reviewed the final version and made the necessary changes, approved the submitted version, and agreed to be accountable for the content of the work.

## Footnotes

1
https://cutadapt.readthedocs.io/en/stable/

2
ftp://ftp.ensembl.org/pub/release-101/fasta/rattus_norvegicus/dna/

3
http://www.bioconductor.org/packages/release/bioc/html/ballgown.html

4
https://sciex.com/products/software/xcms-plus-software
