# Supplementary material for: Multi-omics analysis reveals GABAergic dysfunction after traumatic brainstem injury in rats
Source: Front Neurosci. 2022 Nov 23;16:1003300. doi: 10.3389/fnins.2022.1003300 (PMC9726735; doi:10.3389/fnins.2022.1003300)
Supplement: Supplementary file 4 [file Data_Sheet_4.docx]

Supplementary Material


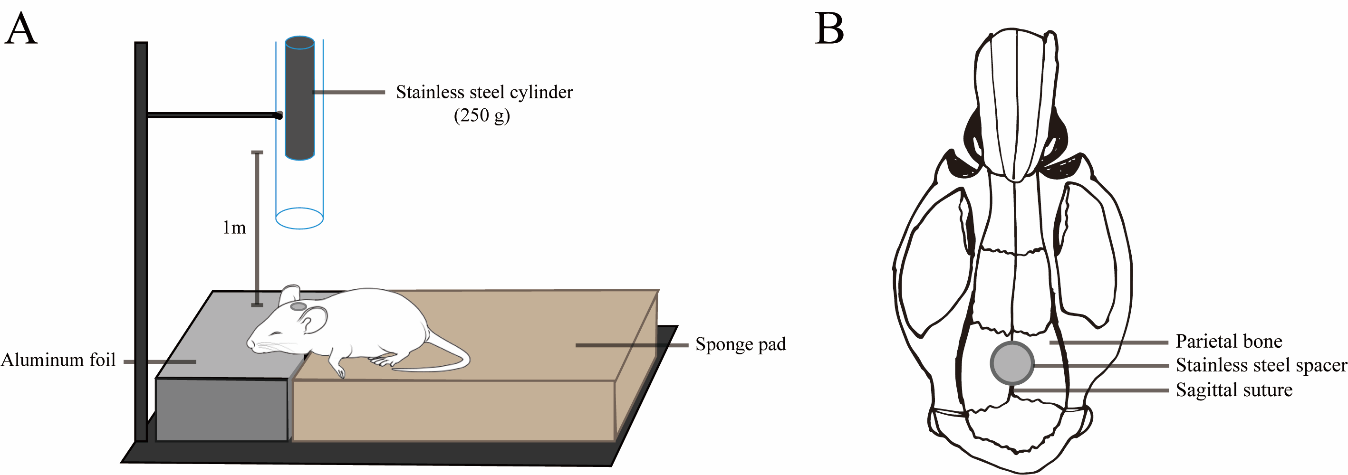


**Figure 1.** The schematic diagram of the mechanical impacting method. (A) Mechanical impacting devices. (B) Location of impact.


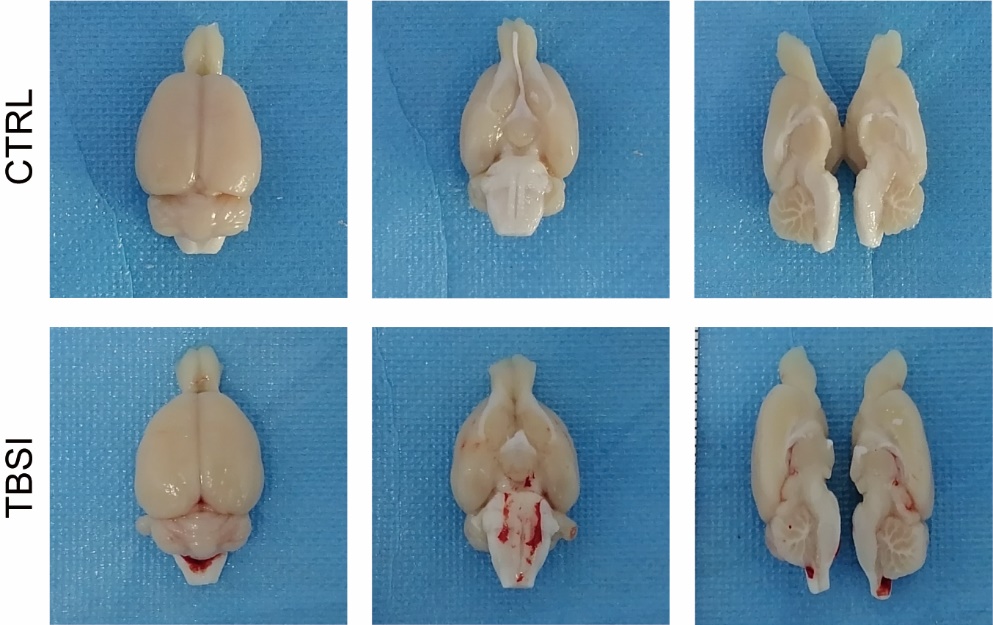


**Figure 2.** Anatomical view of the brain tissues of rats.


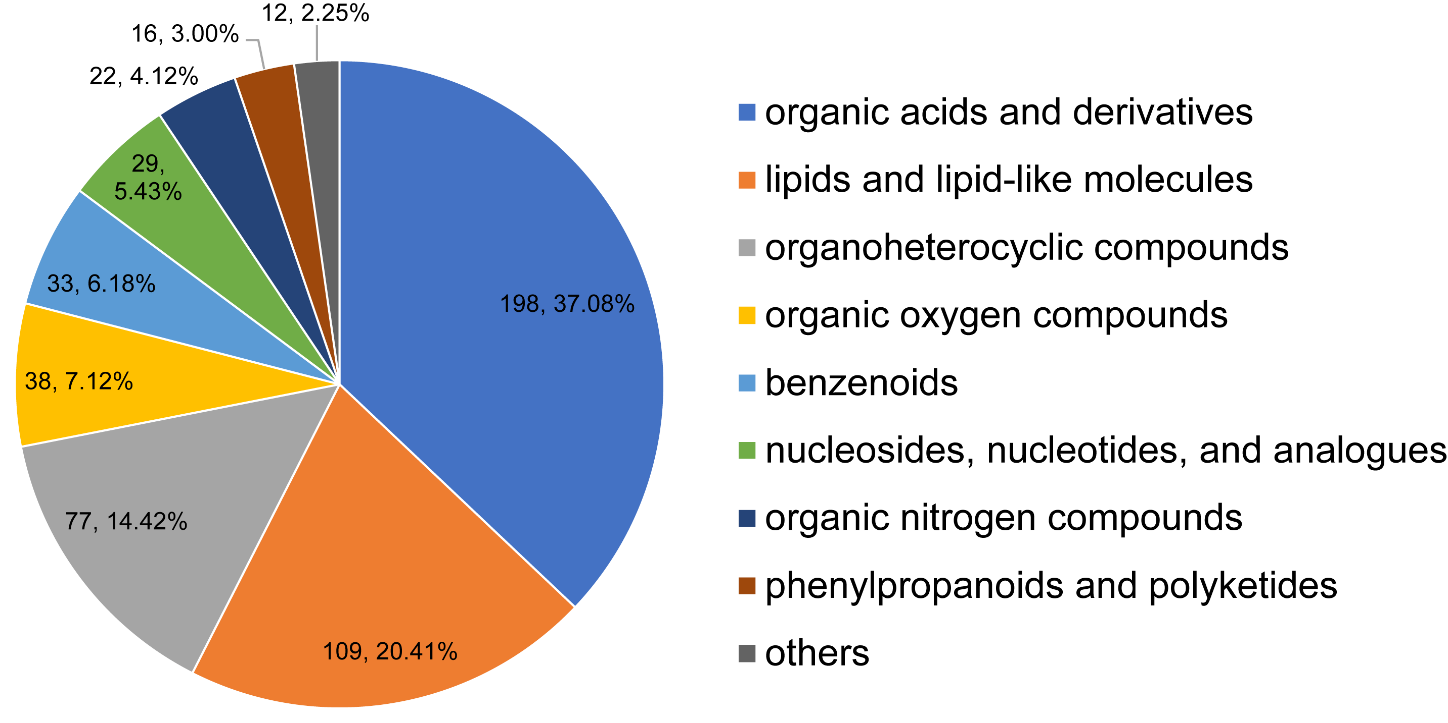


**Figure 3.** Distribution of metabolites.


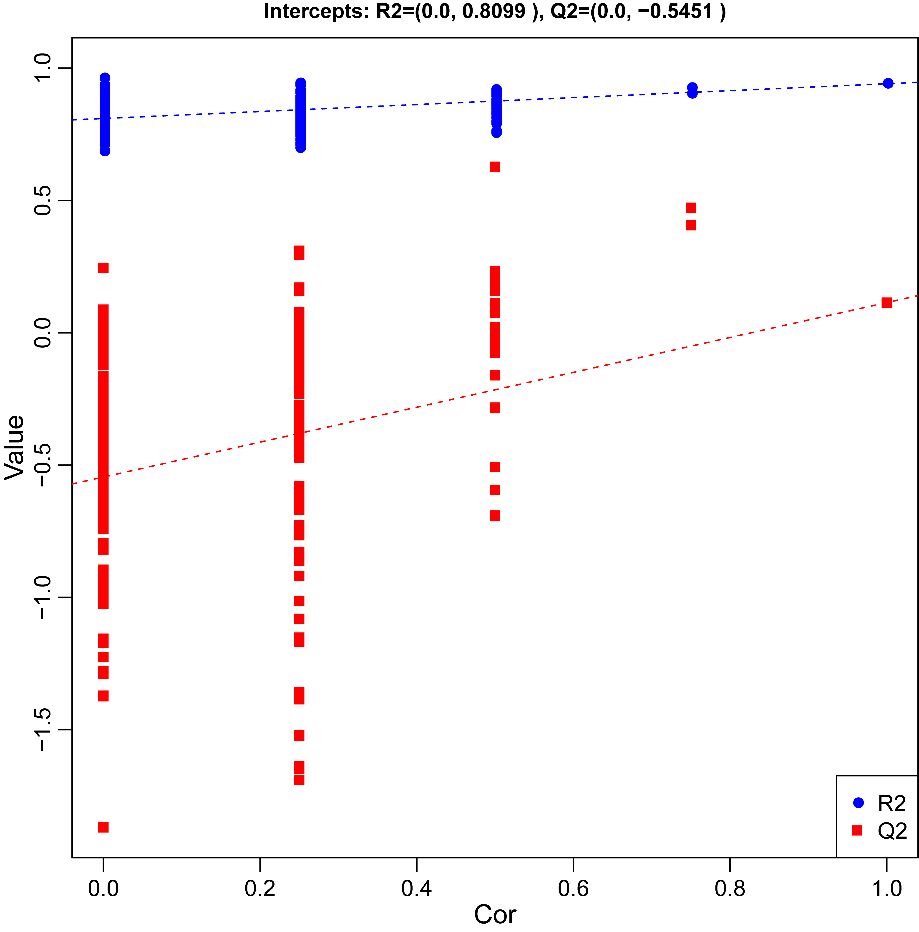


**Figure 4.** Model validation of PLS-DA with the replacement test.
